# Investigation of Distortion, Porosity and Residual Stresses in Internal Channels Fabricated in Maraging 300 Steel by Laser Powder Bed Fusion

**DOI:** 10.3390/ma18051019

**Published:** 2025-02-25

**Authors:** Bruno Caetano dos Santos Silva, Bruna Callegari, Luã Fonseca Seixas, Mariusz Król, Wojciech Sitek, Grzegorz Matula, Łukasz Krzemiński, Rodrigo Santiago Coelho, Gilmar Ferreira Batalha

**Affiliations:** 1 SENAI Innovation Institute for Forming and Joining of Materials, University SENAI CIMATEC, Av. Orlando Gomes, 1845, Salvador 41650-010, Brazil; bruno.silva@fieb.org.br (B.C.d.S.S.); bruna.callegari@fieb.org.br (B.C.); lua.seixas@fieb.org.br (L.F.S.); 2 Department of Mechatronics and Mechanical Systems Engineering, Polytechnic School of Engineering, University of Sao Paulo, Av. Prof. Mello Moraes, 2231, Sao Paulo 05508-030, Brazil; gfbatalh@usp.br; 3 Department of Engineering Materials and Biomaterials, Faculty of Mechanical Engineering, Silesian University of Technology, 44-100 Gliwice, Poland; mariusz.krol@polsl.pl; 4 Scientific and Didactic Laboratory of Nanotechnology and Materials Technologies, Silesian University of Technology, 44-100 Gliwice, Poland; wojciech.sitek@polsl.pl (W.S.); grzegorz.matula@polsl.pl (G.M.); lukasz.krzeminski@polsl.pl (Ł.K.); 5 Post-Graduation Program MPDS/GETEC/MCTI, University SENAI CIMATEC, Av. Orlando Gomes, 1845, Salvador 41650-010, Brazil

**Keywords:** maraging 300 steel (18Ni300), laser powder bed fusion (LPBF), internal channels, distortions, porosity, residual stresses

## Abstract

The use of parts containing internal channels fabricated by laser powder bed fusion (LPBF) in maraging steel is gaining attention within industry, due to the promising application of the material in molds and forming tools. However, LPBF processing has issues when it comes to unsupported channels, leading to defects that can result in a limited performance and shortened component life. The present study aims to provide a detailed evaluation of the metallurgical effects arising from the LPBF printing of channels made of maraging 300 steel. The results show that channel distortion occurs due to the lack of support, associated with increased roughness at the top part of the channel profile caused by partial melting and loosening of the powder. Statistical analyses showed that distortion is significantly affected by channel length. A high level of porosity derived from a lack of fusion was observed in the region above the channel and was attributed to layer irregularities caused by the absence of support, with a predominance of large and irregular pores. Residual stresses, always of a tensile nature, present a behavior opposed to that of distortion, increasing with increases in length, meaning that higher levels of distortion lead to an enhanced effect of stress accommodation/relief, with porosity having a similar effect. All these phenomena, however, did not seem to affect crystallographic orientation, with a nearly random texture in all cases, most likely due to the energy input used and to an optimized laser scanning strategy. These findings are vital to increase the amount of attention paid towards the design of internal channels, especially with those with the purpose of coolant circulation, since distortions and poor surface finishing can reduce cooling efficiency due to a defective fluid flow, while porosity can have the same effect by hindering heat transfer. Residual stress, in its turn, can decrease the life of the component by facilitating cracking and wear.

## 1. Introduction

Maraging steels are the object of substantial industrial interest due to their unique combination of outstanding strength and excellent toughness, in addition to their high dimensional stability and good weldability [1]. Regarding maraging steels processed by additive manufacturing (AM), on one hand, the configuration of martensite in iron–nickel laths makes them relatively tough, meaning that they possess a high capacity to resist cracking during rapid solidification, which is characteristic of AM processes [2]. On the other hand, such rapid cooling leads to the formation of fine cellular/dendritic grains, contributing to strength increases [3]. For this reason, there is an ever-growing interest in the processing of this steel class by technologies such as laser powder bed fusion (LPBF) and directed energy deposition (DED).

One of the most prominent applications of maraging steels is in the manufacturing of molds and tools for the hot stamping process [4,5]. In this sense, the use of additive manufacturing is useful for improving the design of internal cooling channels in these tools, allowing for the development of the so-called conformal channels. These closely follow the geometry of mold cavities, yielding benefits such as a higher cooling efficiency and better thermal homogeneity in the processed component [4,6]. The use of conformal channels can reduce processing times by up to 70% and minimize distortions in the formed part due to the improved heat distribution [7]. However, the fabrication of internal channels by AM, specifically by LPBF, brings along several challenges, e.g., dimensional stability, surface roughness, residual stress and geometrical integrity. In many cases, there is a limitation from the use of support structures inside these channels, which amplifies shape and surface deviations associated with the lack of support [8,9]. All these factors are critical, since they can lead to a decrease in the cooling efficiency of additively manufactured channels [6,9]. Furthermore, it is suggested that issues such as a reduction in cooling efficiency due to the effect of poor surface finishing and distortion on flow [10], stress corrosion cracking and erosion arising from the continuous fluid flow through these channels and failure due to thermomechanical fatigue caused by repeating heating/cooling cycles can reduce both the performance and lifetime of tools fabricated by AM [11,12,13].

Several studies have explored the additive manufacturing of internal channels without support structures. Baier et al. [14] investigated the LPBF of a thin-walled cylindrical object containing sections with varying diameters in an aluminum alloy, with a focus on the effects of geometry on roughness, porosity and distortion. Belloli, Demir and Previtali [9] evaluated the printing of channel prototypes with circular and diamond profiles in maraging steel by LPBF. They developed a model to predict and compensate for channel distortion and stress based on the variation of design parameters (diameter, thickness, wall inclination). Grosjean et al. [13] analyzed the effect of contour strategies on residual stress and mechanical properties, especially fatigue, in maraging steel channels produced by LPBF. However, no experimental correlation was found considering other relevant features, such as microstructure, porosity and texture. Li et al. [15] developed a model to predict and compensate for distortions in channels fabricated by LPBF in a Ti-6Al-4V alloy and, in their work, shape deviations were attributed to a tendency of the material to warp outwards above the channel. Unsupported channels manufactured in AlSi10Mg alloys [16], Ti-6Al-4V alloys [17,18] and 316L stainless steel [19] using LPBF have been studied, providing useful insights about the effects of process parameters, such as scanning angle, scanning speed, laser power and energy input, on distortion and roughness. Additional studies, e.g., those by Cortina et al. [20], Asnafi et al. [4], Muvunzi et al. [21] and Pujante et al. [22], investigated the use of additive manufacturing to print full hot stamping tools or tool prototypes for stamping tests. These works were focused on analyzing features such as thermal distribution and heat transfer in tools and stamped sheets as a function of the design of the cooling system, with little or no attention paid to the detailed metallurgical effects of AM that led to the observed results.

The aim of this work was to conduct a thorough characterization of prototypes of circular channels with no support structure fabricated in maraging 300 steel by LPBF. This characterization will help us to understand the correlations between the lack of support, associated distortions and relevant metallurgical aspects of the material, such as microstructure, porosity, residual stress and texture. Such comprehension, in its turn, can serve as support for the creation of adequate channel designs, involving strategies to mitigate the aforementioned issues, such as loss of cooling efficiency and a decrease in tool life due to wear, corrosion and stress.

## 2. Materials and Methods

### 2.1. Specimen Design

**Figure 1:**
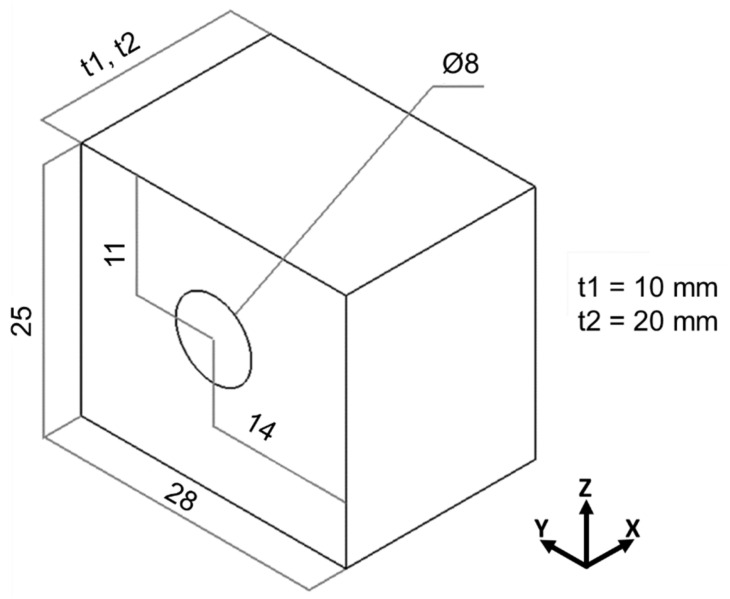
Geometry of part manufactured by LPBF (dimensions in millimeters).

The design of the channel specimens for LPBF is shown in Figure 1. Channels were manufactured through thickness in both cases (“t1” and “t2”). The variation in thickness was based on the findings of Belloli, Demir and Previtali [9], who observed that the channel’s length can affect the intensity of distortions generated in its circumference. Nevertheless, changes were made to the specimen’s geometry, aiming to abide by design guidelines for the intended application of the manufactured channels in tools for hot stamping of metal sheets [23]. For the same reason, a diameter of 8 mm was set for the channel, based on future application requirements while respecting the limitations of the LPBF technique, and a distance of 11 mm from the center of the channel to the top surface of the specimen was used considering the specified dimensions for hot stamping tool design, sized to meet the mechanical and heat transfer requirements.

### 2.2. Material

The feedstock used was an 18Ni300 maraging steel (1.2709) powder supplied by Böhler (Vienna, Austria) with particle size distributions of “D10” = 18–24 μm, “D50” = 29–35 μm and “D90” = 42–50 μm and a minimum bulk density of 3.5 g/cm^3^ [24]. Table 1 presents the chemical compositions of the raw material provided in the datasheet and measured in the printed steel by inductively coupled plasma optical emission spectrometry (ICP-OES) in an Oxford Instruments Foundry-Master Pro spectrometer (Abingdon, UK). Before the analysis, the specimen was mechanically ground with #180 sandpaper and subsequently cleaned with water and isopropyl alcohol. The values shown correspond to the average of five measurements. The composition of the processed material, despite showing small variations with respect to the one provided by the manufacturer, is sufficiently close to the specified values.

### 2.3. LPBF Processing

**Figure 2:**
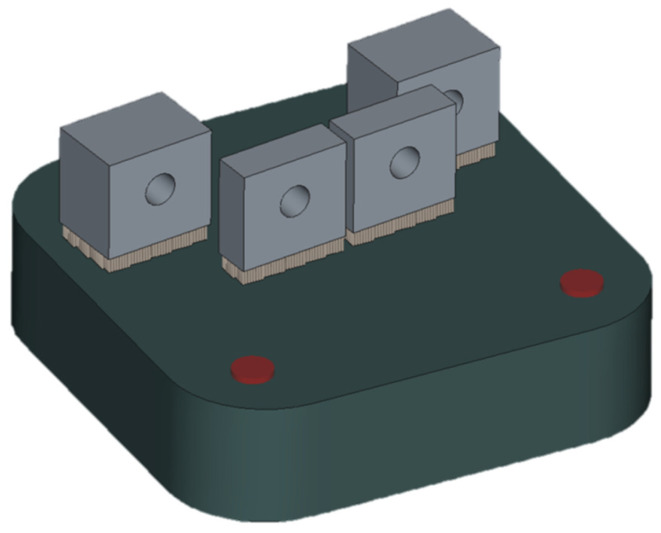
Build plate design for the LPBF printing of channel specimens.

Channel specimens were additively manufactured by LPBF in a Renishaw AM125 (Wotton-under-Edge, UK) printer with a printing volume of 120 × 120 × 125 mm. A rotation of 7° around the *Z*-axis was used to avoid contact between the printed parts and the material coater, which could lead to printing defects (Figure 2). The printing parameters described in Table 2 are the same as those considered optimal in the study by Król et al. [25] for the maraging steel supplied by the same company and printed in the same machine. These parameters fall within the optimal processing window suggested by Guo et al. [1] for LPBF of maraging steel. Preheating was used with the objective of reducing distortions and residual stresses [26,27,28].

It is worth mentioning that, although two “t1” and two “t2” specimens were printed, only one “t1” and one “t2” specimen were used for the present analyses. The remaining ones were heat-treated, and their results will be the object of another publication. In all the characterization steps listed below, except for tomography, as will be explained, one “t1”specimen and one “t2” specimen were analyzed.

### 2.4. Characterization

#### 2.4.1. Three-Dimensional Scanning

Three-dimensional scanning was carried out to evaluate the dimensional and geometric accuracy of the manufactured channels. A Zeiss ATOS Q 12M 3D (Oberkochen, Germany) scanner equipped with LED lighting, a fringe projection system with a Blue Light Equalizer and two cameras with resolution of 12 megapixels were employed. A measuring volume of 100 mm (100 × 70 mm^2^ projection) was used. Post-processing involved the generation of a triangular mesh, characterized by finer triangulations in regions exhibiting increased geometric complexity, and measurements of dimensional and geometric parameters. All steps were carried out in GOM Inspect Pro 2022 software.

#### 2.4.2. Density Measurement

For reference, density measurements were carried out in cubes of 8 × 8 × 8 mm^3^ using a Gehaka DSL910 density meter (São Paulo, Brazil) with millesimal precision. Samples were previously ground with #180 sandpaper on all faces to ensure geometric accuracy. The weight of samples was initially measured in dry conditions, followed by weighing in a basket immersed in ethyl alcohol with density of 0.78999 g/cm^3^.

#### 2.4.3. Computed Tomography

Tomographic imaging was conducted to characterize porosity size, morphology and distribution. A Nikon XT H 225 ST 2x industrial CT system (Minato, Japan) with a tungsten anode and a Gadox detector was used. Analyses were performed with 360° rotation, in full shift mode, with a voltage of 90 kV, spatial resolution of 5 µm and a source-to-detector distance of 815 mm. In total, 4476 projections were generated, with 4 frames per projection, resulting in 71,616 frames. Data were processed using Nikon CTPro 3D and VolumeGraphics VGStudio MAX 2023 software (VGEasyPore plugin and VGDefX/Only Threshold algorithms). The distributions of pore diameter, volume, sphericity and compactness were analyzed. For this analysis, specimen “t2” was selected due to its capacity to provide a larger analytical volume, thereby facilitating the acquisition of more representative and statistically significant results. This choice is critical for enhancing the reliability of the findings and ensuring that the data reflect the underlying phenomena with greater accuracy.

#### 2.4.4. X-Ray Diffraction

To identify the phases present in the microstructure of maraging steel and to measure residual stresses arising from the LPBF process, the X-ray diffraction (XRD) technique was used in a four-circle PANalytical MRD-XL diffractometer (Almelo, the Netherlands) with a molybdenum anode. Conventional scans for phase identification were performed within a 2-theta (“2θ”) range from 15° to 45°, with step size of 0.05°, a time per point of 40 s and a beam size of 5 × 5 mm. The scanning interval was sufficient to acquire four peaks associated with the ferritic/martensitic phase and four associated with the austenitic phase. Phase analysis was performed only in specimen “t2”, chosen for the same reasons as for tomography, and based on the hypothesis that channel length would not affect significantly the amount of retained/reversed austenite, if present.

Residual stress measurements were performed in both specimens using the sin^2^psi method [29,30], with the same step size and time per point, using seven evenly spaced values of the psi (“ψ”) angle varying between 0 and 71.5°, with a 2-theta scan between 52.5° and 58°. For these measurements, a high multiplicity ferrite peak of the {123} family was chosen. The tabulated X-ray elastic constants (“S_1_” and “S_2_”) used in this work are provided in Equations (1) and (2), where “E” and “ν” are the Young’s Modulus and Poisson’s ratio, respectively.

(1) S1=−vE=−0.890927 MPa−1

(2) $$\frac{1}{2}S_{2}=\frac{1+\nu }{E}=4.85619\;{\mathrm{MPa}}^{-1}$$

**Figure 3:**
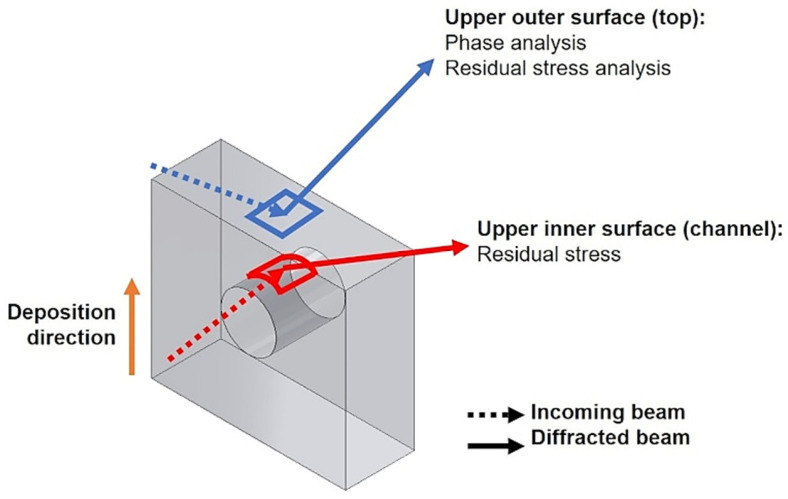
Out-of-scale schematic indication of the locations of incidence of the X-ray beam for identifying phases and measuring residual stresses.

Residual stresses were measured at the central positions of the outer top surface of the specimens, exactly above the channel, and on the inner top surface of the channel, while scanning for phase identification was performed only on the outer top surface, as shown in Figure 3. Attention was also given to the stress state on the upper surface of the specimen for two reasons: (1) porosity distribution in the region below may influence residual stress levels, and (2) in hot stamping conditions, this surface will be in contact with heated blanks and thus constantly exposed to wear and thermomechanical fatigue. After scans, stress calculations were performed in a self-developed module running in the MathWorks MATLAB R2015a software (Natick, MA, USA).

#### 2.4.5. Microstructural Analysis

**Figure 4:**
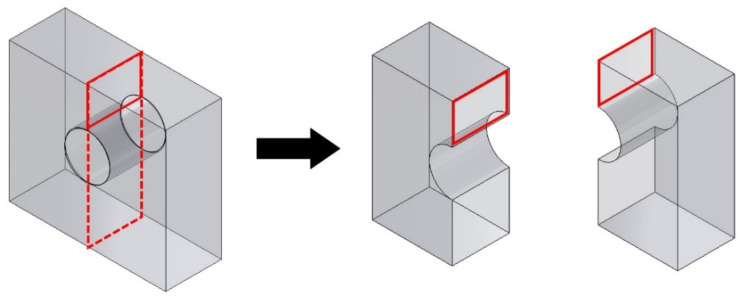
Indication of the region of interest in channel specimens for SEM analyses.

Scanning electron microscopy (SEM) was performed in a JEOL JSM-6510 LV microscope (Akishima, Japan) with a tungsten filament using a secondary electron signal, with a beam acceleration voltage of 20 kV, beam size of 20–40 (arbitrary unit) and working distance of 10 mm, on the plane of specimens cut as shown in Figure 4. Both samples were embedded in bakelite, ground with progressively finer sandpaper up to 1200 µm, polished with 1.0 and 0.3 μm alumina suspensions and etched with Nital 2% to reveal the microstructure.

Electron backscatter diffraction (EBSD) analyses were carried out on the same surface indicated in Figure 4 for both specimens. Maps were acquired using an Oxford X-Max detector (Abingdon, UK) coupled to a Thermo Fisher Scientific Quanta 650 field emission gun SEM (Waltham, MA, USA) operated at 20 kV. For EBSD, the final surface preparation included vibratory polishing using a 40 nm silica suspension until a condition with optimal surface relief was obtained. Maps were acquired at regions close to the outer specimen surface and to the upper surface of the channel with step sizes of 0.08 µm for high-magnification maps and 0.45–0.58 µm for low magnification. Images were processed in Oxford AZtecHKL software (https://nano.oxinst.com/products/aztec/aztechkl (accessed on 19 February 2025)) for data cleaning and Kernel average misorientation (KAM) calculation.

## 3. Results and Discussion

### 3.1. Distortions

**Figure 5:**
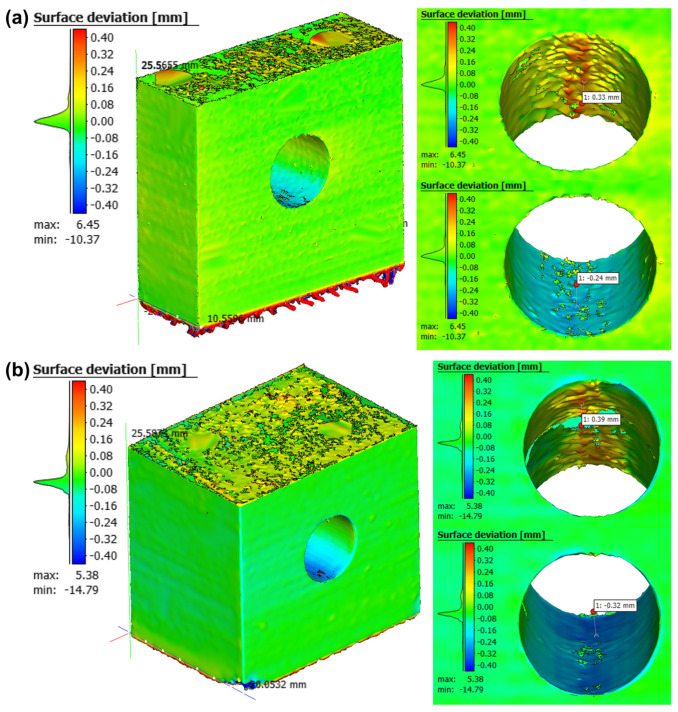
Surface deviations between as-printed (3D scans) and designed samples: (**a**) t1; (**b**) t2.

The comparison between the diameters of the designed and printed parts (measured by 3D scanning) is shown in Figure 5 and summarized in Table 3. It is possible to observe irregularities at the top of the channels, caused by the lack of support, leading to the highest average levels of deviation in this region, thereby contributing to variations in relation to the designed diameter value. Specimen “t1” presented a higher deviation, which is in accordance with previous findings indicating that, as the channel length increases, deviation decreases [9].

Considering that the data groups of the channel diameter results have approximately equal variances, which was verified through a Levene homogeneity test (Table 4), a one-way ANOVA was applied with the Fisher method to evaluate the effect of channel length on the diameter. Through this analysis of variance, it was verified that the effect of length on the diameter has statistical significance, which can be seen in Table 5. Thus, it is expected that further length variations will also generate variations in the channel diameter.

### 3.2. Porosity

**Figure 6:**
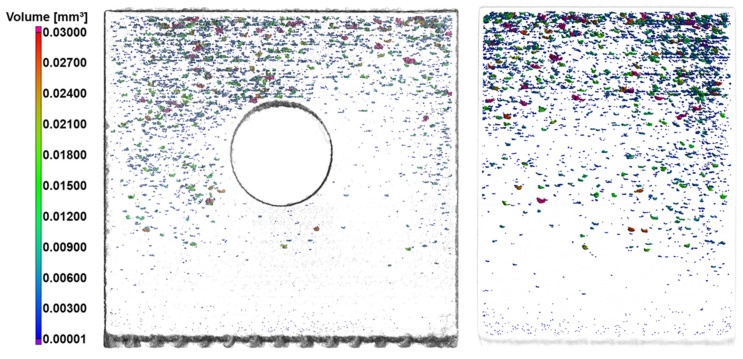
Porosity distribution in specimen “t2”, with volumetric pore classification.

The distribution of porosity in specimen “t2” is shown in Figure 6. An evident concentration of porosity is observed in the upper half of the part, above the channel. The accumulation of pores in this region can be associated with irregularities in layers, due to the lack of support [14]. It has already been shown, e.g., by Song et al. [31], Bai et al. [32] and Shange et al. [33], that defects generated on the surface of molten layers considerably increase the formation of pores, being affected by excessive energy in the molten pool or insufficient support of the deposited layers. The distortion results obtained from the 3D scans are indicative of irregularities between layers, which can be held responsible for the intensified appearance of porosity in regions above the channel. It is also possible to observe different morphologies of pores, with both small and large pores, as well as spherical and irregular ones. The region with the greatest formation of larger and irregular pores is the top of the part, suggesting a cumulative effect of layer irregularity during specimen buildup, while the base presents mostly smaller, spherical pores.

**Figure 7:**
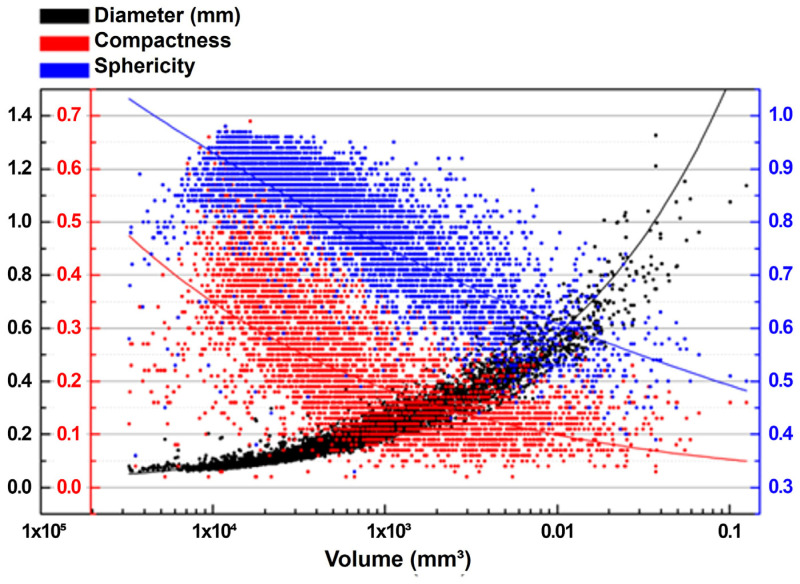
Distributions of pore diameter, compactness and sphericity as functions of pore volume in specimen “t2”.

The distributions of pore sphericity, compactness and diameter as functions of volume are shown in Figure 7. Sphericity is a measure of how close a body mathematically resembles a perfect sphere, being the main means of evaluating pore shape [34]. This parameter corresponds to the ratio between the surface area of a sphere with the same volume and the actual pore surface area [35]. The compactness of a pore is defined as the ratio between the volume of the pore and the volume of a sphere with a diameter equal to the maximum diameter that circumscribes the pore [35]. Generally, the greater the volume or diameter of the pores, the lower the sphericity and compactness. Pore sphericity and compactness variations are usually ascribed to different pore formation mechanisms, i.e., larger and irregular pores are frequently associated with a lack of fusion, while smaller and more spherical pores are associated with keyhole effects or gas entrapment [32,36]. However, pores can also be associated with voids generated by poor compaction [37], either due to covering problems or irregularities in the material, which is the most likely case in the present study, since the lack of support can cause irregularities in deposited layers.

Moreover, it is possible to observe a large variation in the compactness and sphericity results. In other studies, this behavior has been attributed to the high volumetric energy density [38] and the reuse of powder recycled from previous processing [39]. In this study, a relatively high energy density, of around 160 J/mm^3^, was applied, and recycled powder was used. However, density measurements in cube samples provided an average density of 7.9526 g/cm^3^ for the printed maraging steel, with a deviation of −1.82% with respect to the density of 8.10 g/cm^3^ reported in the material datasheet [24]. This translates to a relative density of 98.18%, which can be considered high (>97%), according to Król et al. [25], meaning that the intensified porosity observed in the channel specimens results primarily from the printing of unsupported channel features, not from processing.

**Figure 8:**
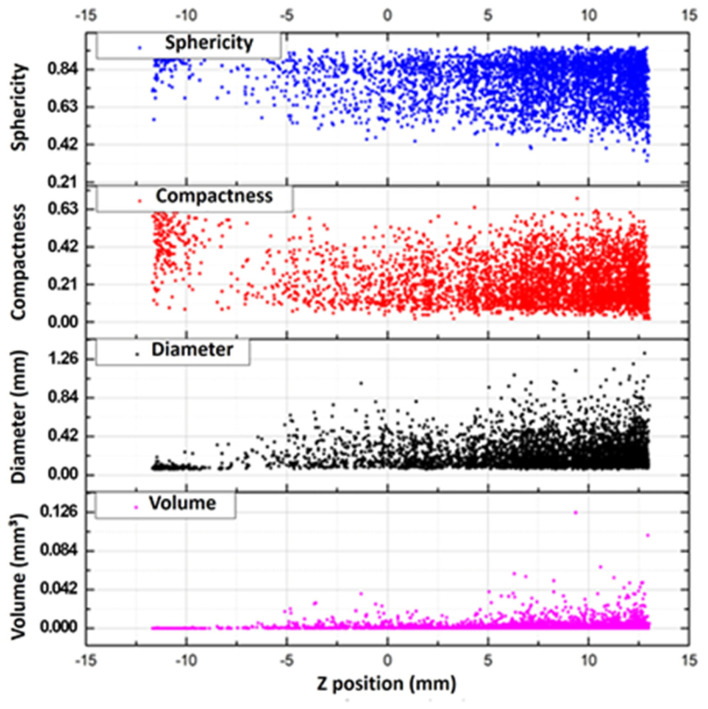
Distributions of pore sphericity, compactness, volume and diameter along the height of specimen “t2”.

Distributions of pore sphericity, compactness, volume and diameter along the height of the evaluated part are shown in Figure 8. The results show a greater homogeneity of sphericity and, to a lower extent, compactness at the base of the piece. In these regions, the pores have smaller volumes and diameters that gradually increase along the height of the piece, with a greater concentration of large pores with a high dispersion of compactness and sphericity at the top. A minimum pore diameter of about 0.85 mm and a maximum of about 1.32 mm were identified. One factor that may have affected this distribution could be, again, the lack of support in regions above channels, as already observed in other studies, where the formation of pores is intensified by irregularities formed in layers with little support [33,40].

**Figure 9:**
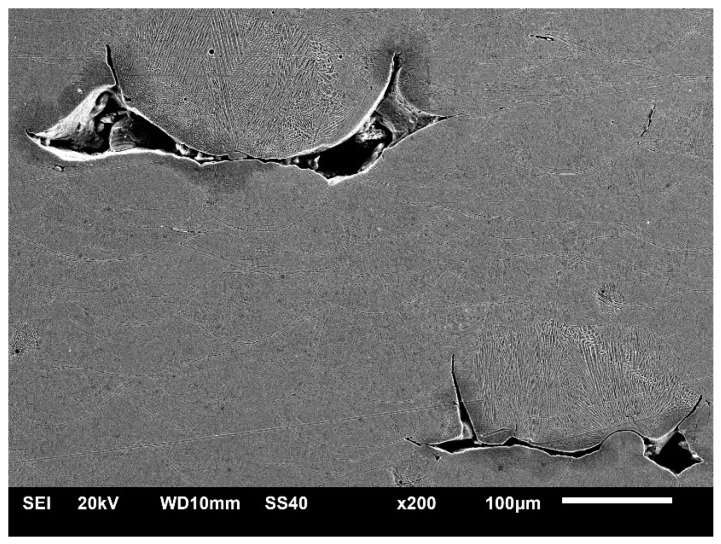
Low-magnification SEM image showing the aspect of pores observed in specimens.

A representative image of the predominant porosity aspect in the upper part of specimens can be observed in Figure 9, which shows a low-magnification image of the pores between melt pools, characteristic of lack of fusion. As discussed above, the formation of pores due to a lack of fusion is related to voids or regions with partially molten material, due to insufficient energy deposition into the layers. Pores have an irregular shape and are formed between regions of consecutive passes [41,42]. These observations are in accordance with the tomography results, which indicate the presence of coarser and more irregular pores in this region, starting from the channel up to the top surface of the piece. Understanding the emergence of defects such as porosity and how to mitigate them is necessary not only to improve the structural quality of parts manufactured by LPBF, but also to ensure the better performance of hot working tools. The thermal conductivity of metal parts manufactured by AM can be reduced by volume defects, as in the case of cracks or pores, which reduce the density of the material [43,44].

### 3.3. Residual Stresses

**Figure 10:**
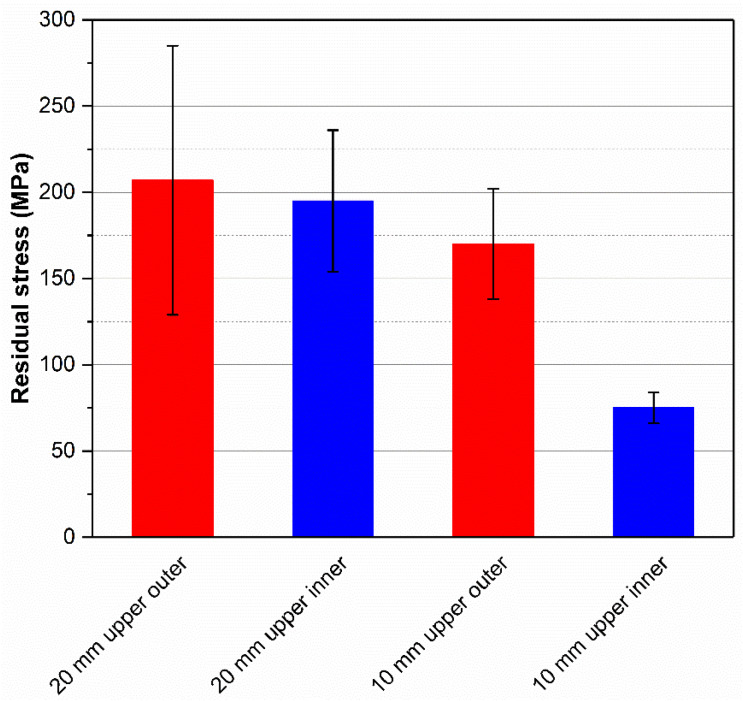
Summary of residual stress values, with their respective errors.

The residual stress values are summarized in Figure 10. A tensile stress state is seen in all assessed regions, which can be ascribed to the “warping” tendency of the unsupported material deposited by LPBF [6,15]. These findings go against those of Belloli, Demir and Previtali [9], who observed compressive stresses in maraging 300 steel channels. Grosjean et al. [13] obtained a stress profile going from compressive on the specimen surface to tensile in the vicinity of the channel; their study, however, involved a change in the deposition strategy for the final layers to improve surface finishing. Moreover, measurements were carried out on the surface parallel to the printing direction (XZ/YZ), not on the channel and specimen’s top surfaces (XY), as in the present case, meaning that the stresses were measured in different directions. General trends include stress reduction from specimen “t2” to “t1” (decreasing thickness), and lower stress levels on the upper inner surface of the channel as compared to the upper outer surface of the specimen. However, these differences are almost negligible due to the relatively high associated errors, which can be caused by the roughness of surfaces produced by LPBF, especially inside the channel, likely due to the coarseness of prior austenite grains with columnar growth and the eventual textures that can also arise from the process. The most notable difference is between channels with different lengths (“t2” vs. “t1”). The fact that residual stresses are lower for the specimen “t1” than for “t2”, especially inside the channel, while the surface deviation results present an opposite behavior, being higher for the channel in “t1”, might suggest that the lack of support structures allows higher levels of distortion to take place during processing, with a consequent effect of stress accommodation and relief. In fact, the magnitudes of residual stress (pre-relief) and distortion (post-relief) are directly correlated, as shown by Mugwagwa, Yadroitsev and Matope upon analysis of LPBF-printed cantilevers made of maraging steel 300: the higher the stress level in the cantilever before cutting, the higher the resulting distortion after cutting [45]. It is worth mentioning that the presence of porosity can produce a stress relief effect and reduce distortions as well [46]. Negligible stress variation on the outer surface of samples indicates that the channel region is the most sensitive one to the effects of LPBF processing, while the stress state on the outer surface most likely results from a combination of rapid cooling due to contact with open air and to the intensified porosity concentration in the final building layers.

### 3.4. Microstructure

**Figure 11:**
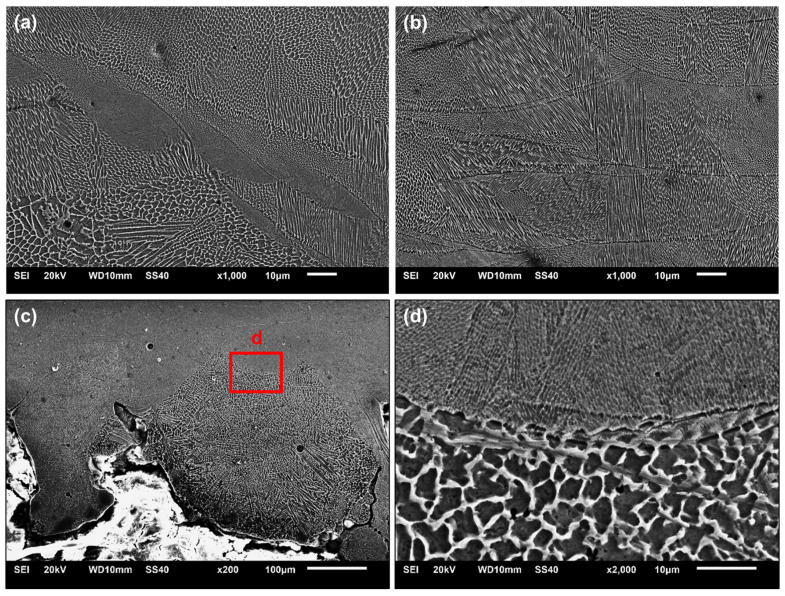
Representative SEM images of the bulk microstructure of as-printed maraging 300 steel: (**a**) specimen “t1”; (**b**) specimen “t2”; (**c**) upper inner surface of the channel in specimen “t2”; (**d**) detail from “d” showing differences between consolidated and overhanging features on the surface of the channel.

Representative images of the microstructure of maraging steel as printed, in the plane perpendicular to the deposition direction, are shown in Figure 11a,b. The microstructure is composed of cellular and columnar grains of the martensitic phase with different degrees of refinement. As the X-ray diffraction analysis did not indicate the presence of austenite in this condition, it is understood that the enrichment of martensitic cell boundaries with elements such as Mo, Ni and Ti is not sufficient to promote the stabilization of austenite [47]. The morphology of the grains that form inside the melt pool basically depends on the relationship between the thermal gradient and the solidification rate: the greater this relationship, the greater the tendency for cell growth, and the lower the ratio, the greater the tendency towards columnar growth [41,48]. Although columnar growth is more common in the printing direction, variations in orientation, morphology and refinement are associated with local variations in cooling and heat extraction rates in different directions, which strongly depend on process variables [49,50]. The cellular and columnar structures grow epitaxially across the boundaries of the melt pool, as solidification occurs in a direction perpendicular to the boundary of the melt pool, associated with the direction of the maximum thermal gradient during solidification [1,51]. These structures are consistent with what was identified in other studies focused on the LPBF of maraging steel, such as those by Mutua et al. [52] and Tan et al. [53].

Detailed images of the surface conditions close to the upper inner surface of the channel are shown in Figure 11c. The surface is extremely irregular, including the presence of partially molten powder particles resulting in large droplets, whose degree of microstructural refinement differs from that of the consolidated printed material to a large extent (Figure 11d), which can be regarded as an overhanging feature caused by the lack of support. The same aspects were observed regardless of channel length. Factors such as a lack of support and the staircase effect can lead to heat accumulation or thermal gradients that intensify surface irregularities and roughness, but also cause distortions and changes in the channel profile [17]. Similar aspects were observed by Yang et al. [16] on the upper surface of AlSi10Mg channels produced by LPBF, by Tian et al. [18] in the overhanging tilted features of an LPBF-processed Ti-6Al-4V alloy and by Belloli, Demir and Previtali [9] in maraging steel itself.

**Figure 12:**
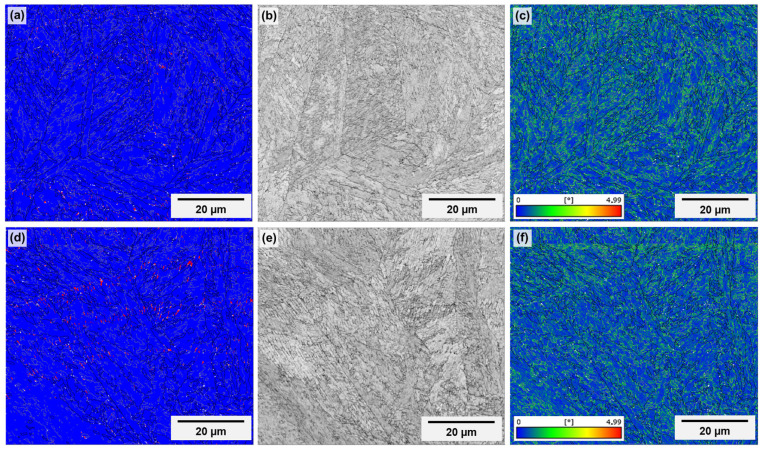
High-magnification EBSD maps of specimen “t1” showing phase distribution (**a**,**d**), pattern quality (**b**,**e**) and Kernel average misorientation (**c**,**f**) of the upper outer specimen surface (**a**–**c**) and of the upper inner channel surface (**d**–**f**). In phase maps, blue corresponds to ferrite and red to austenite. The building direction corresponds to the height of the images.

**Figure 13:**
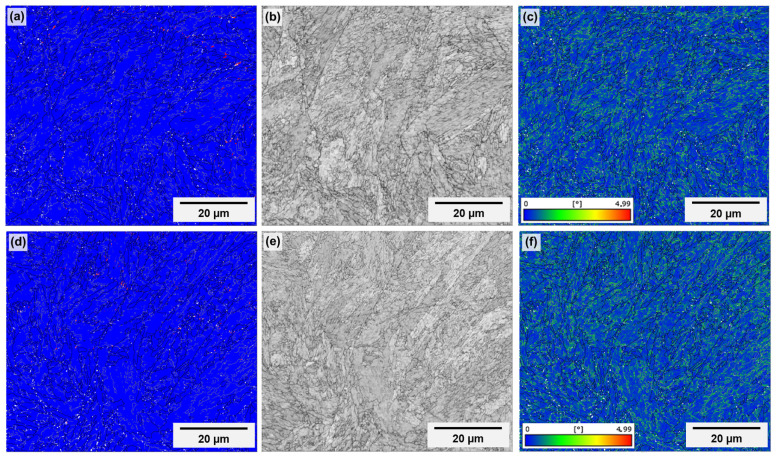
High-magnification EBSD maps of specimen “t2” showing phase distribution (**a**,**d**), pattern quality (**b**,**e**) and Kernel average misorientation (**c**,**f**) of the upper outer specimen surface (**a**–**c**) and of the upper inner channel surface (**d**–**f**). In phase maps, blue corresponds to ferrite and red to austenite. The building direction corresponds to the height of the images.

**Figure 14:**
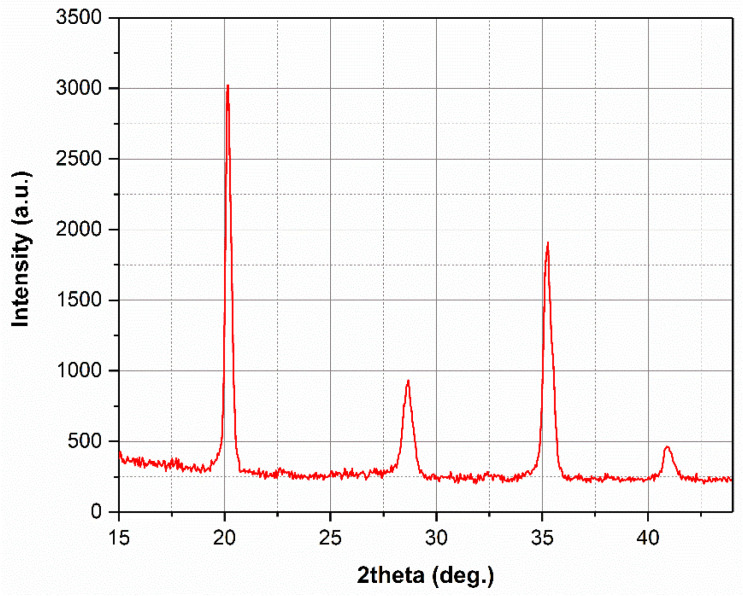
Diffractogram of as-printed maraging steel.

High-magnification EBSD data for specimens “t1” and “t2” are presented in Figure 12 and Figure 13, respectively. It is important to emphasize that the maps above the channel were acquired in the vicinity of features regarded as partially melted particles with coarse microstructures, to focus on regions that are representative of the bulk itself. Regarding the phase maps (images “a” and “d”), the blue color corresponds to ferrite and the red one, to austenite. In all cases, virtually no austenite (<1.0%) is present, corroborating the XRD results. A slight tendency for higher amounts of austenite above the channel and in specimen “t1” (0.9% in “t1” and 0,2% in “t2”, against 0.4% on the top surface of “t1” and 0.1% on the top surface of “t2”) exists. However, it is not possible to draw any tangible conclusion, since these amounts are too low to be considered as separate from measurement and post-processing errors. In Figure 14, the diffractogram obtained for the as-printed condition is shown. It reinforces that, after printing, the microstructure is predominantly martensitic and, if any austenite fraction was present, it would be in amounts below the detection limits of the present X-ray diffraction configuration (around 2.0 vol%). In fact, it would be more likely to detect austenite in maraging steel in higher quantities after heat treatment. The austenite observed in the treated condition is commonly called reversed austenite, and is mainly favored during the aging stage of the material [52,54], due to local enrichment of molybdenum, titanium and, mainly, nickel in martensitic grain boundaries [49,55].

To compare the quality of both regions, two parameters were chosen: the pattern quality (images “b” and “e” in Figure 12 and Figure 13), and the Kernel average misorientation (KAM) (images “c” and “f” in Figure 12 and Figure 13). Pattern quality gives off information such as defect density or recrystallization state because the quality of the diffraction pattern is directly affected by the degree of deformation of the material, being lower in more defective microstructures. KAM is useful to assess the dislocation density because it is particularly sensitive to low-angle misorientations and is calculated as the average misorientation of a specific data point with its nearest neighbors [56]. The images, however, show no appreciable differences between both regions in the same specimen and between the same regions in both specimens.

**Figure 15:**
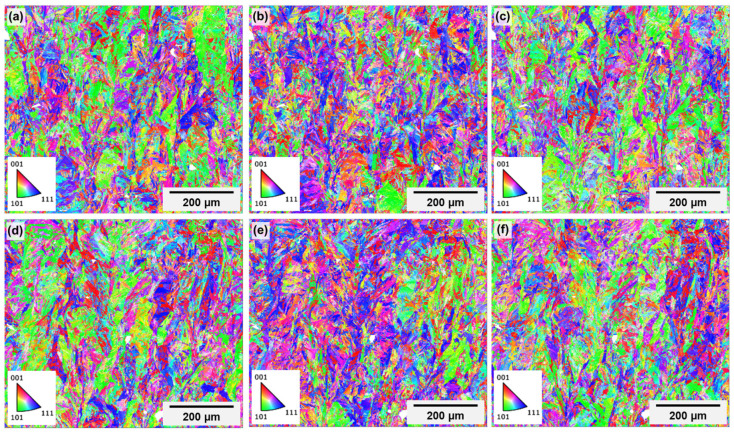
Low-magnification IPF maps in the X (**a**,**d**), Y (**b**,**e**) and Z (**c**,**f**) directions of the upper outer specimen surface (**a**–**c**) and of the upper inner channel surface (**d**–**f**). The building direction corresponds to the height of the images.

**Figure 16:**
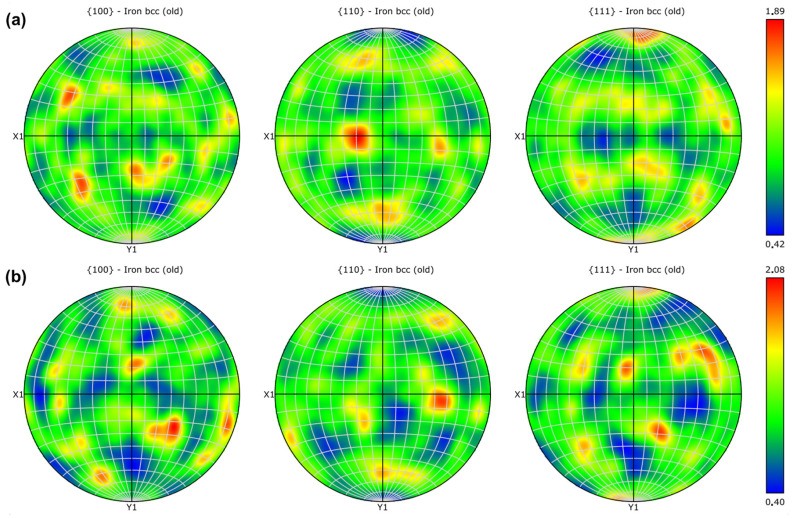
Pole figures: (**a**) upper outer specimen surface; (**b**) upper inner channel surface.

Low-magnification maps were acquired to provide information about crystallographic texture with better grain statistics. Because the high-resolution maps indicated no tangible differences between the different regions and specimens, just the low-magnification maps of “t2” are presented. Inverse pole figure (IPF) maps of both regions are shown in Figure 15, with all three main directions as a reference (bearing in mind that the *Z*-axis of the image corresponds to the Z-direction of specimens—see Figure 4), and the respective pole figures (PF) are presented in Figure 16. The two regions present similar texture characteristics. It is possible to observe a columnar character of the prior austenite grains that form first during cooling, with a growth direction parallel to the building direction. However, the martensitic laths inside these prior grains show a quasi-random orientation, given the relatively low texture indexes, although these indexes are slightly higher in the upper inner surface of the channel. This may be due to the 67° rotation between the layers used during printing, which is known to alter heat flux direction and to reduce texture intensity [49,53]. Moreover, Kannan et al. [57] demonstrated that the energy input of LPBF processing affects the crystallographic texture in maraging steels, going from near random at lower input to a <001> cube texture as the energy increases. The authors also observed that, as heat input increased, although the crystallographic texture became stronger, porosity was minimized, and hardness was maximized. The heat input range tested in their work—20–85 J/mm^3^—was much lower than the one used in the present work, but the powder supplier was also different, and no information about the equipment used for printing was given. Therefore, no direct comparison is possible. However, the fact that, in this work, a nearly random texture associated with a high level of porosity was observed could mean that decreasing the heat input may help to minimize porosity at the expense of microstructural isotropy.

## 4. Conclusions

The present work evaluated the printing of internal channels in maraging 300 steel via LPBF, with a focus on the metallurgical effects of the process for this type of feature. A thorough characterization framework was utilized, involving techniques such as 3D scanning, computed tomography and crystallographic analyses. The proposed study provides knowledge on the possible effects arising from the printing of channels without support structures, which is of the utmost importance for consideration when designing components for AM containing intricate cooling system designs, as is the case for tools used for hot stamping. The main conclusions were as follows:

- Distortion in channels is primarily due to the lack of support structures, decreasing with increasing channel length. Statistical analyses showed that channel length exerts a significant influence on diameter variation. Distortion might affect the cooling efficiency of the channel due to its likely effects on fluid flow.
- High levels of porosity were observed above the channels, which can be attributed to layer irregularities caused by the absence of support. The porosity is characterized by large, irregular pores, which are more prevalent in the upper regions of specimens. The presence of porosity might reduce heat transfer between the channel and the part’s surface, also affecting the process’s efficiency when it comes to the cooling of forming tools.
- Residual stresses are tensile in nature, which can be ascribed to a “warping” tendency, and increase with channel length, as opposed to distortion, suggesting that higher levels of distortion contribute to a stress relief effect, with the same effect also being attributed to the presence of pores. On the top surface of specimens, stresses are higher than at the top of the channel. This effect may be critical because it makes the part prone to cracking and wear during operation. However, such effects might be at least partially mitigated by heat treatment, which will be the object of future study.
- Regardless of the specimen region, the microstructure is composed of cellular and columnar grains of the martensitic phase with different degrees of refinement, with no detectable presence of retained or reversed austenite. The upper inner region of the channel has an extremely irregular surface composed of partially melted particles. Such irregularity suggests an elevated roughness, which can also affect fluid flow and cooling efficiency.
- The crystallographic texture of martensite is nearly random, possibly due to a combination of the energy input and an optimized laser scanning strategy.

## Figures and Tables

**Table 1:** Chemical compositions (wt%) of the steel powder according to Böhler [24] and measured in the bulk material after LPBF processing. Balance: Fe.

Source	Ni	Co	Mo	Ti	Al	Mn	Si	C	P	S
Supplier	18.0	9.30	4.90	1.10	0.15	≤0.15	≤0.10	≤0.03	≤0.01	≤0.01
Measured (average)	18.60	8.44	4.70	0.95	0.16	0.03	<0.005	<0.005	<0.005	<0.005
Measured (error)	0.23	0.16	0.14	0.06	0.06	<0.00	<0.00	<0.00	<0.00	<0.00

**Table 2:** LPBF parameters used to manufacture channel specimens in maraging 300 steel.

Parameter	Value
Laser wavelength	1070 nm
Laser power	200 W
Laser focus size	0.07 mm
Scanning speed	350 mm/s
Layer thickness	30 µm
Hatch spacing	120 µm
Atmosphere	Argon 4.6
Preheating	80 °C
Printing direction	Horizontal
Scanning strategy	67° rotation between subsequent layers

**Table 3:** Comparison between designed and printed diameters.

Parameter	t1 = 10 mm	t2 = 20 mm
Designed diameter (mm)	8.0	8.0
Printed diameter (mm)	7.871	7.990
Deviation (%)	−1.6%	−0.1%

**Table 4:** Homogeneity of variance test (Levene) for channel diameter data.

Parameter	F-Value	df 1	df 2	*p*-Value
Diameter (mm)	4.70	1	7	0.067

**Table 5:** One-way ANOVA (Fisher) for the effect of channel length on diameter.

Parameter	F-Value	df 1	df 2	*p*-Value
Diameter (mm)	22.5	1	7	0.002

## Data Availability

The original contributions presented in this study are included in the article. Further inquiries can be directed to the corresponding author.

## Abbreviations

The following abbreviations are used in this manuscript:

AM	Additive manufacturing
EBSD	Electron backscatter diffraction
KAM	Kernel average misorientation
LED	Light emitting diode
LPBF	Laser powder bed fusion
SEM	Scanning electron microscope/microscopy
XRD	X-ray diffraction
